# Effectiveness and side effects of dimethyl fumarate in multiple sclerosis after 12 months of follow up: An Iranian clinical trial

**Published:** 2019-10-07

**Authors:** Mohsen Foroughipour, Sahar Gazeran

**Affiliations:** Department of Neurology, School of Medicine, Mashhad University of Medical Sciences, Mashhad, Iran

**Keywords:** Multiple Sclerosis, Magnetic Resonance Imaging, Dimethyl Fumarate

## Abstract

**Background:** Multiple sclerosis (MS) is a neurologic disorder with a considerable global burden. During the last decades, some pharmaceutical treatments have been approved for patients with MS. Dimethyl fumarate (DMF) is one of these drugs which has been reported to have early promising results in recent studies, but the efficacy of this drug in patients with MS is still being studied in different parts of the world. In the present study, we evaluated the effectiveness of DMF therapy on reducing relapses, lesions, and disability in Iranian patients with MS.

**Methods:** The present single-arm before-after study was approved by the Ethics Committee of Mashhad University of Medical Sciences, Mashhad, Iran [Iranian Registry of Clinical Trial (IRCT) code: IRCT20190121042439N1]. Every patient who was diagnosed with relapsing MS was considered eligible to enroll in the present clinical trial. Before receiving DMF therapy, the baseline liver function tests and complete blood count were obtained from all individuals. Also, a baseline brain magnetic resonance imaging (MRI) was obtained and Expanded Disability Status Scale (EDSS) was documented from all patients. After receiving 240 mg DMF twice daily for 12 months, the laboratory and imaging measurements as well as EDSS were repeated. Furthermore, the total number of relapses within the study period was recorded. Satisfaction with DMF treatment was determined by answering a yes-no question.

**Results:** A total number of 50 patients enrolled in the study and most of them were female (80%). There was a significant decrease in EDSS score and gadolinium (GD)-enhancing lesions after the study period (P < 0.001 for each). Moreover, the attacks significantly dropped after the study period (P < 0.001) and 86% of patients were satisfied with their treatment.

**Conclusion:** The findings of this study showed that 240 mg DMF administered twice daily can effectively reduce disability and provide satisfaction within the first year of therapy in patients with MS.

## Introduction

Multiple sclerosis (MS) is a common neurologic disorder with considerable burden in most countries. Individuals with MS experience difficulty in their social relationships and employment and also face decreased life quality. The number of individuals with MS has increased from 2.1 million to 2.3 million in 5 years since 2008.^1^ Regarding the extent of MS care facilities and developments in therapeutic approaches, the MS Disability-Adjusted Life Year (DALY) rate has been decreased in both developed and developing countries.^2^ The early diagnosis of this demyelinating disease is beneficial for patients and results in a better therapeutic response.^3^ Treatment of MS can be divided into different stages including treating acute phase and using disease-modifying drugs (DMDs). While the cornerstone of MS treatment is avoidance of factors triggering or exacerbating the disease, some acute relapses can be treated by steroids. Disease modifying therapies can be used in different types of MS. DMDs are usually used to manage relapsing forms and can improve quality of life in different ways. Among DMDs, dimethyl fumarate (DMF) is an immunomodulatory drug, which has been used in MS. This drug has provided promising results in treating psoriasis. DMF is hydrolyzed to monomethyl fumarate (MMF), which delivers its immunomodulatory effect. The main mechanism of action of DMF is based on induction of both adaptive and innate immune systems. Recently, the beneficial effect of DMF in patients with MS has been demonstrated in two large clinical trials, including CONFIRM [Comparator and an Oral Fumarate in Relapsing-Remitting MS (RRMS)] trial and DEFINE (Determination of the Efficacy and Safety of Oral Fumarate in RRMS) trial.^4^^,^^5^ However, some mild to moderate side effects have been reported for DMF in patients with MS, including mostly gastrointestinal (GI) symptoms and to some less extent progressive multifocal leukoencephalopathy (PML).^4^^,^^5^ According to the substantial increase in use of DMDs over the past 2 years, the efficacy and tolerability of such agents in patients with MS is yet highlighted.^6^ The aim of the present clinical trial was to evaluate the efficacy of DMF in Iranian patients with RRMS who were irresponsive to first-line treatment.

## Materials and Methods

The present single-arm before-after clinical trial was approved by Ethics Committee of Mashhad University of Medical Sciences, Mashhad, Iran, and was registered at Iranian Registry of Clinical Trial (IRCT) database (IRCT code: IRCT20190121042439N1). The study population was chosen from patients who were referred to Comprehensive MS Center, which is under supervision of the Mashhad University of Medical Sciences. All patients diagnosed with RRMS who were within the age range of 15 to 50 years and were willing to participate in the study were considered eligible to enroll in this study. An informed consent was obtained from all patients prior to participation in the study. Exclusion criteria for this study were pregnancy, liver dysfunction, lymphopenia, and presentation of adverse effects resulting in the discontinuation of the treatment. A demographic questionnaire including the number of attacks in the previous year was filled by all patients. All patients received interferon beta (INFβ), 39 patients (78%) received INFβ-1a and 11 patients (22%) received INFβ-1b before shifting to DMF. The reason for shifting to DMF was patient dissatisfaction regarding the complications and regular injections. Before DMF prescription, baseline liver function tests as well as complete blood count were obtained from all patients. Furthermore, a baseline brain magnetic resonance imaging (MRI) was obtained from all patients. The Expanded Disability Status Scale (EDSS) was obtained from all patients by the same researcher before the initiation of DMF treatment. Oral DMF was prescribed at the dose of 240 mg twice daily to all patients. The liver function tests and complete blood count were assessed every 3 months for one year. Furthermore, each patient was checked for possible complications during the DMF treatment period. All patients were informed about the possible complications in each visit and were requested to contact the researcher if any complication occurred during the one-year study period. Any patient who had severe drug complication and could not tolerate the drug was planned to switch to another therapy under supervision of a neurologist and was excluded from the study. After 12 months, all patients underwent brain MRI and filled EDSS. All patients were asked to report the total attacks within the study period and also report their satisfaction with the new treatment by answering a yes-no question at the end of study period. 

Data were analyzed using SPSS software (version 20, IBM Corporation, Armonk, NY, USA). Continuous data were checked for normality using the Shapiro-Wilk test. Continuous data were presented using mean and standard deviation (SD), while frequency and percentage were used for categorical variables. The Wilcoxon signed-rank test was used to compare continuous variables between measurements. The chi-square and Fisher’s exact tests were used to compare the distribution pattern of categorical variables between measurements. The level of statistical significance was set at P < 0.05.

## Results

**Figure 1:**
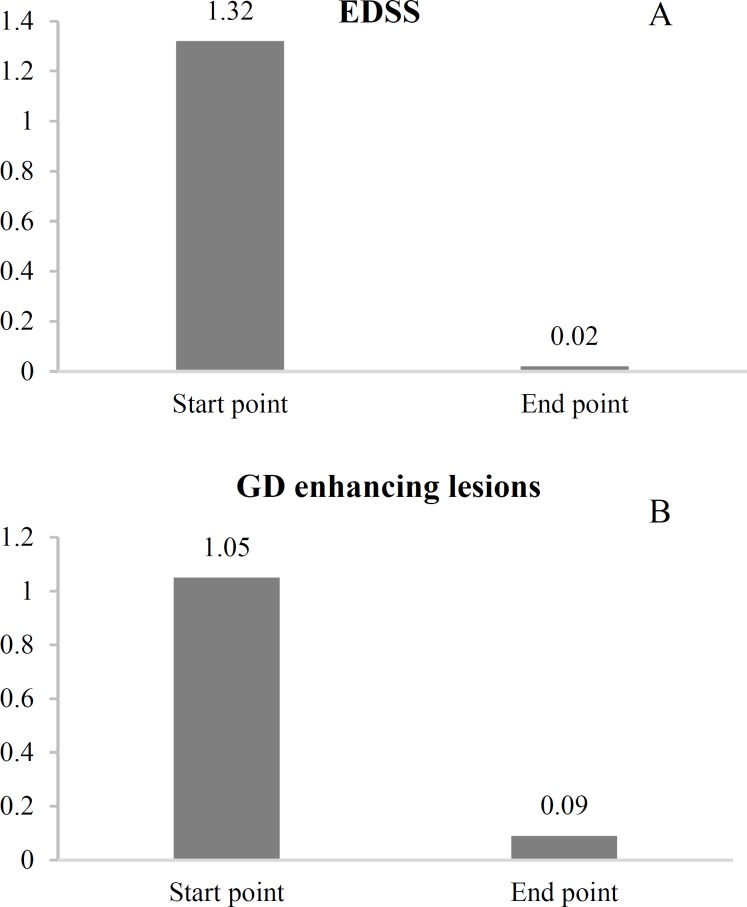
The mean Expanded Disability Status Scale (EDSS) score (A) and gadolinium (GD)-enhancing lesions (B) before and after treatment

A total number of 50 patients with relapsing MS enrolled in the present study. Most of the participants were female (80%) and most of the patients (34%) were between 25 to 30 years old. The disease duration was between 24 and 48 months in 54% of the patients followed by less than 24 months in 40% and more than 48 months in 6% of the patients. The Wilcoxon test revealed significant decrease in EDSS score and gadolinium (GD)-enhancing lesions at the end of the study period (P < 0.001 for both) (Figure 1, A and B). The mean EDSS score decreased after 12 months from 2.82 to 2.53 (P < 0.001). The Wilcoxon test showed that the number of relapses significantly dropped after the study period (mean number of attacks at baseline and at the end of the study were 1.32 ± 0.55 and 0.02 ± 0.14, respectively, P < 0.001). Most of the patients were satisfied with their treatment (86%). Complications occurred in 25 (50%) patients during the study duration. Among the 25 patients with complications, 17 (68%) had only one complication, 4 patients (16%) had 2 complications, while 3 and 4 complications were each reported by 2 patients (8%). The frequency of treatment complications is summarized in table 1. There was no significant difference in the distribution pattern of treatment complications between genders (Table 1).

## Discussion

Regarding the importance of managing the relapsing MS, this clinical trial tried to assess the efficacy and outcomes of treatment with DMF in patients with relapsing MS. According to our results, administration of DMF for patients with relapsing MS is beneficial in reducing the attack rate and EDSS score as well as reducing GD-enhancing lesions. A recent German study by Braune et al. demonstrated that using DMF was more effective than other first-line agents including INF, glatiramer acetate, and teriflunomide in real world.^7^ Ontaneda et al. evaluated patients with MS receiving DMF and fingolimod.^8^

**Table 1:** Frequency of treatment complications as per gender

**Complication**	**Male**	**Female**	**P**	**OR**	**95% CI for OR**		**Phi**
	**n (%)**	**n (%)**			**Lower**	**Upper**	
Nausea	3 (20)	12 (80)	0.348	2.180	0.378	12.572	0.113
Abdominal pain	1 (10)	9 (90)	0.571	0.334	0.022	5.041	-0.058
Flushing	2 (25)	6 (75)	0.310	2.332	0.238	22.824	0.138
Diarrhea	1 (25)	3 (75)	0.464	2.590	0.152	44.185	0.093
Liver enzyme disruption	0 (0)	2 (100)	0.737	< 0.001	< 0.001	-	0.560
Lymphopenia	0 (0)	0 (0)	-	-	-	-	-

OR: Odds ratio; CI: Confidence interval

They demonstrated that switching to DMF reduced the risk of relapse by 32% compared to fingolimod.^8^ Furthermore, a French study reported similar findings for DFM compared to teriflunomide.^9^ Moreover, postmarketing evaluation in other countries, including Italia, have also proved the short-term safety and effectiveness of DMF. It was previously stated that EDSS score was 0.08 ± 0.44 per year and patients with higher EDSS tended to have higher clinical and radiological activity.^10^ In line with the findings of our study, Kresa-Reahl et al. also demonstrated that patients with relapsing MS benefited from DMF. DMF has resulted in lower relapse rate and improved patient-reported outcomes.^11^ Gold et al. evaluated the effect of different doses of DMF in patients with MS. They stated that delayed-release DMF could successfully improve clinical outcomes based on EDSS scale. The mean EDSS scores in the control group, as well as BID and TID administered DMF groups were 2.2, 2.1, and 2.0, respectively.^12^ By taking 240 mg DMF twice daily, our patients experienced a reduction in EDSS score from 1.32 to 0.22. Moreover, D’Amico et al. demonstrated that those patients who were treated with DMF had lower EDSS score compared to those who were treated by teriflunomide.^13^ According to the approved safety of DMF in MS, our Iranian study could also successfully demonstrate the efficacy of DMF. 

However, still some studies provided different results including Kalincik et al.,^14^ which included a larger sample size and longer follow-up duration than our study. Kalincik et al. demonstrated that among oral immunotherapies for RRMS, fingolimod was associated with less relapse rate and discontinuation in contrast to DMF and teriflunomide. The cumulative hazards of disability accumulation and improvement were not different among DMF and fingolimod groups.^14^ It has been previously noticed that for patients who were using first-line treatment and injectable agents, shifting to DMF would be a safe choice regarding the tolerability and safety issues and that DMF might improve annual relapse rate.^15^ However, another large-scale study suggested that for those who were switching from self-injectable drugs to DMDs, fingolimod was superior to DMF.^16^ Moreover, two recent meta-analysis studies showed no difference between fingolimod and DMF regarding relapse and disability, although the follow-up periods in the two meta-analyses were shorter than the follow-up duration in the study by Kalincik et al.^14^ which followed their patients for 2.5 years.^17^^,^^18^ These controversial results highlight the fact that still some studies with longer follow-up period and large sample size may be required to draw a firm conclusion about the superiority of DMF over other drugs including fingolimod. Regardless of the efficacy and superiority of DMF, the side effects and complications are the other concerns which have been addressed in the literature. According to the possible relation between development of cancer and using immunosuppressant drugs, risk management plans are mandatory for using some of these agents including fingolimod, teriflunomide, and DMF.^19^ Serious safety concerns have been identified in less than 2% of patients with RRMS who received DMF.^13^ GI events are common complication of DMF.^20^ Our study also demonstrated that GI complications were major concerns for those patients with MS who were using DMF. Although using bismuth subsalicylate did not affect GI events overall, it could affect the incidence and severity of some complications, including diarrhea and flatulence.^20^ Our population demonstrated that DMF complications mostly included nausea, abdominal pain, flushing, diarrhea, and abnormalities in liver function tests. Another Asian study revealed almost the same results. They stated that relapse in DMF group (29.00%) was lower than placebo group (47.00%). Flushing (14.25%), GI events (20.36%), and infections (27.48%) as well as cardiovascular (2.40%) and hepatic events (9.16%) were considered as common adverse effects. During their second phase of study, both of study groups received daily DMF (240 mg BID). During this phase, the incident of adverse effects, including flushing, GI events, and increase in liver function tests as well as skin events remained stable in both groups.^21^

Regarding the neuroimaging findings in our study, it has been suggested that DMF can reduce MRI activity. Gold et al. demonstrated that different doses of DMF could have different effects on GD lesions. According to their results, GD lesions were higher in BID administration of DMF compared to TID administration.^12^ While in our study we used 240 mg of DMF twice daily, we achieved a significant reduction in GD score from 1.05 to 0.09 in one year. Saida et al.^22^ evaluated the efficacy of delayed-release DMF (240 mg BID) in East Asia and demonstrated the favorable use of DMF in patients with RRMS, which was similar to the findings of our study. In their study, DMF could successfully reduce new GD lesions from baseline to week 24 by 75% and reduce the number of relapses by 42% over a 24-week period.^22^

## Conclusion

Relapsing MS is a chronic neurologic disorder that can be managed without significant complications if appropriate treatment is identified. DMF is a newly-used drug for treatment of relapsing MS and is still being studied for long-term effects in various clinical trials. The results of the present clinical trial showed that treatment of relapsing MS with DMF would provide decreased attack rate and GD lesions. Moreover, most of the patients were satisfied with their treatment and EDSS score was significantly reduced without any serious complications, which may result in termination of DMF therapy.

## Notes:

**How to cite this article:** Foroughipour M, Gazeran S. Effectiveness and side effects of dimethyl fumarate in multiple sclerosis after 12 months of follow up: An Iranian clinical trial. Iran J Neurol 2019; 18(4): 154-8.
